# Identifying skull fractures after head trauma in infants with ultrasonography: is that possible?

**DOI:** 10.1007/s40477-024-00907-7

**Published:** 2024-06-27

**Authors:** Riccardo Filice, Francesca Miselli, Isotta Guidotti, Licia Lugli, Giovanni Palazzi, Alberto Berardi, Lorenzo Iughetti

**Affiliations:** 1 Post-Graduate School of Pediatrics, Department of Medical and Surgical Sciences of the Mother, Children and Adults, University of Modena and Reggio Emilia, 41125 Modena, Italy; 2 Neonatal Intensive Care Unit, University of Modena and Reggio Emilia, 41125 Modena, Italy; 3 PhD Program in Clinical and Experimental Medicine, University of Modena and Reggio Emilia, 41125 Modena, Italy; 4 Department of Medical and Surgical Sciences of the Mother, Children and Adults, University of Modena and Reggio Emilia, 41125 Modena, Italy

**Keywords:** Pediatric, Children, Head trauma, Skull fracture, Ultrasound

## Abstract

Management of pediatric head trauma requires a delicate balance between accuracy and safety, with a dual emphasis on prompt diagnosis while minimizing radiation exposure. Ultrasonography (US) shows promise in this regard. A case study involving a 10-month-old infant with acute right parietal swelling revealed the utility of US in detecting a corresponding hypoechoic lesion, along with an underlying suspected fracture line of the vault and subdural hematoma. Subsequent CT confirmed the fracture, while MRI confirmed the subdural hematoma. At one-month follow-up, MRI demonstrated hematoma reabsorption, while US revealed a bone callus in its advanced phase. Although US is not yet standard practice for pediatric head trauma, its ability to detect fractures in infants suggests its potential role: when a fracture is evident on US, it may serve as an indication to perform neuroimaging. Potentially, adoption of US could contribute to mitigation of children’s exposure to ionizing radiation.

**Supplementary Information:**

The online version contains supplementary material available at 10.1007/s40477-024-00907-7.

## Introduction

Head traumas in children represent a common reason for visits to the Emergency Room (ER). Their incidence varies across countries, with an estimated 47–280 per 100.000 children presenting to the ER each year due to traumatic brain injury worldwide. The risk of skull fracture and brain injury after head trauma is inversely proportional to age: identifying skull fractures in young children is crucial as they are known risk factors for brain injury [1].

Due to its high resolution and ability to detect subtle fractures, Computed Tomography (CT) has become widely utilized for diagnosing (or excluding) skull fractures in children. Currently, head CT is the most common CT scan performed in the pediatric population. However, considering that children are more sensitive to ionizing radiations and have a higher relative risk of cancer, physicians are striving to minimize the use of X-rays and CT scans. Alternative solutions include the development of clinical guidelines to reduce unnecessary exposure, and the adoption of alternative imaging methods including magnetic resonance imaging (MRI) and ultrasonography (US) [2].

US is a noninvasive tool that is superior to CT and MRI in detecting the dura’s status. Moreover, it is preferred over MRI in the ER due to its higher availability. Rabiner et al. stated the following benefits for point-of-care ultrasound (POCUS): (a) early diagnosis of a patient and early consultation; (b) reduction in CT scan request; (c) suitability when access to CT scan is limited; and (d) serving as a triage tool in disaster area with difficult conditions [3].

## Case presentation

**Fig. 1:**
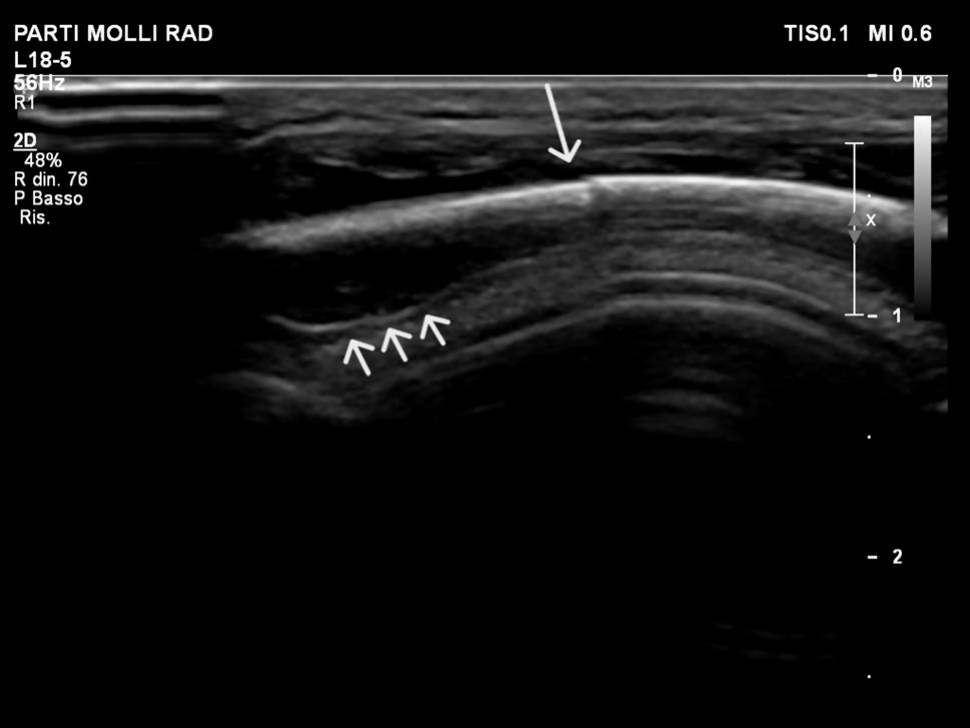
Point-of-care Ultrasound performed using a very-high frequency (18–5 MHz) linear array transducer. The longitudinal view reveals a linear skull fracture (long arrow), accompanied by a small subdural hematoma (short arrows), corresponding to the clinically apparent parietal swelling. The image is obtained in the coronal plane, providing a visualization of the fracture and its spatial relationship with the surrounding tissues

We describe the case of a 10-month-old infant presenting at the ER with an acute parietal swelling over the past 12 h. The medical history revealed no incidents of accidental falls or head trauma. The physical examination revealed a soft, right parietal swelling measuring approximately 2 × 1 cm. The overlying skin was intact, and there was no apparent pain upon palpation. Neurological examination was normal. While awaiting for skull CT in sedation, a cerebral US revealed “a hypoechoic lesion (fluid corpuscular material) at the subcutaneous level corresponding to the swelling, along with a suspected fracture line of the vault and subdural hematoma” (Fig. 1; Online Resource 1).

**Fig. 2:**
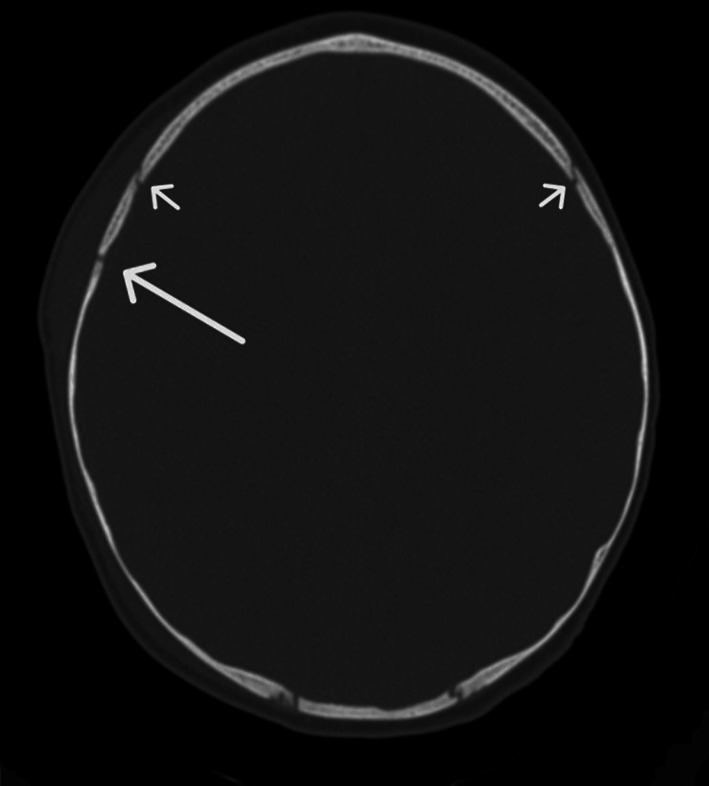
Axial CT scan without contrast of the brain reveals a compound skull fracture (indicated by the arrow) and a hematoma in the right parietal area

These findings were confirmed by a skull CT in sedation that revealed “the presence of hematoma in the right parietal area, a compound fracture of the underlying skull, and an associated layer of subdural hematoma with a maximum thickness of 4 mm along the adjacent convexity.” (Fig. 2).

**Fig. 3:**
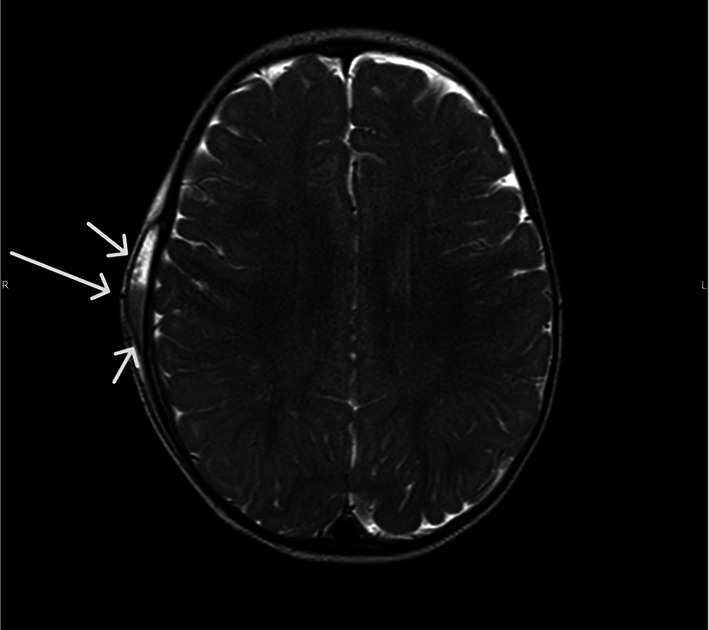
T2-weighted TSE Brain MRI in the transverse plane reveals subdural blood in the right parietal area (short arrows), with minimal imprint on the adjacent parenchyma. A compound fracture of the right parietal cranial theca is also observed (long arrow)

**Fig. 4:**
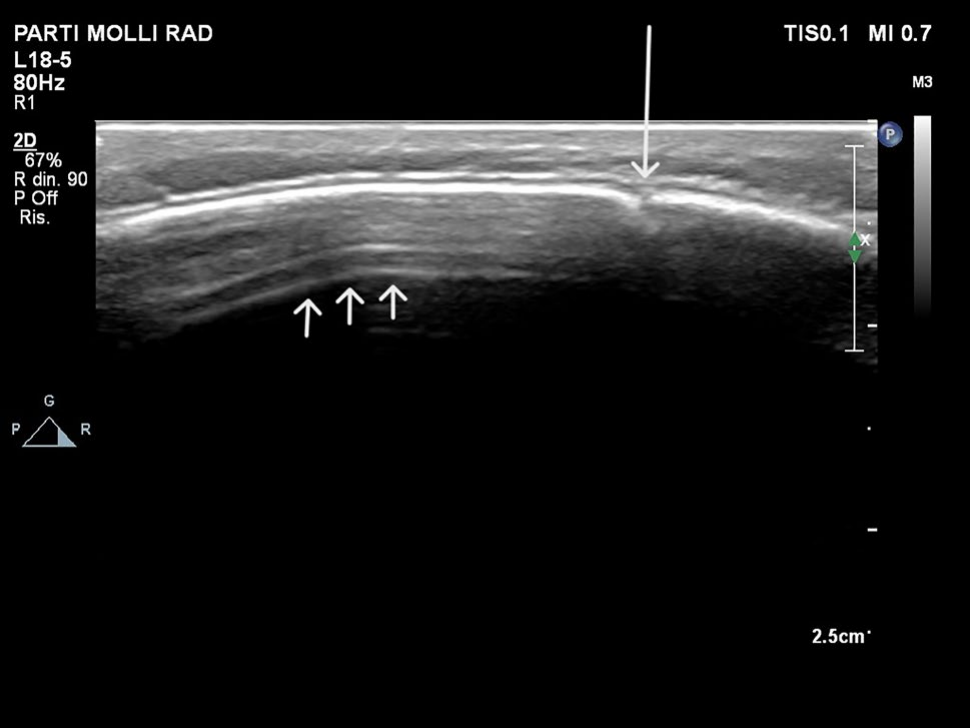
Point-of-care Ultrasound performed using a very-high frequency (18–5 MHz) linear array transducer. An advanced stage of the bone callus is evident. The bone callus (arrow) appears as a hyperechoic line, similar to the surrounding normal bone cortex, but the underlying reverberation artifact (short arrows) are absent. Of note, the aforementioned artifact stops abruptly exactly at the transitional zone from the normal bone cortex to the prominence of the callus

Based on the neuroradiological findings, the neurosurgical consultant recommended in-hospital monitoring for the next 48 h. A brain MRI was also performed, confirming the presence of “subdural blood in the right parietal area, with a maximum thickness of about 3 mm, exerting minimal imprint on the adjacent parenchyma. A compound fracture of the right parietal cranial theca with associated cephalohematoma with posterior blood level was identified.” After demonstrating good general conditions, the patient was discharged with a protective helmet and scheduled for follow-up (Fig. 3). One month later, a follow-up brain MRI revealed reabsorption of the thin right parietal subdural blood layer. On US, we observed the presence of the bone callus in its advanced phase (Fig. 4; Online Resource 2).

## Discussion and conclusions

In current practice, head CT stands as the gold standard diagnostic test for assessing skull fractures and intracranial bleeding subsequent to head trauma. The algorithm outlined in the PECARN guidelines is extensively utilized to determine the necessity of CT scans in children with head trauma [4]. The PECARN study identifies three age-independent predictors, including loss of consciousness, altered state of consciousness (GCS < 15), and high-energy dynamics. Additionally, there are three variable predictors that are age-dependent: changes in behavior, hematoma of the scalp in the 'non-frontal' area, and palpable fracture of the vault in children < 2 years. For older children, repeated vomiting, worsening headache, and suspected fracture of the base are considered as predictors. Subsequently, the patients are categorized into three groups based on the described variables: high risk, requiring immediate CT; low risk, necessitating observation; and moderate risk, where the decision between CT and observation depends on specific clinical features and physician experience.

Although skull US is not yet commonly used and is not included in clinical guidelines, its adoption could potentially assist clinicians in decision-making for cases of moderate risk, potentially reducing the need for CT scans. US can be used to detect skull fractures, particularly in cases where the location of the injury is clear, and the skin is intact. When the fracture is evident on US, it serves as an indication to perform a CT scan to rule out intracranial injuries [5]. Several are the expected advantages of US. First, it can be performed rapidly, enabling earlier detection of a skull fracture as a marker for suspected intracranial injury and facilitating neurosurgical consultation. Second, unlike CT and MRI, US does not require sedation in young children [3]. Finally, and of utmost importance, US has the potential to reduce CT use and minimize ionizing radiation exposure in children. The estimated lifetime risk of cancer from a head CT is substantially higher for children than for adults due to a longer latency period and greater sensitivity of developing organs to radiation. However, it is crucial to note that intracranial injury may occur without skull fracture, and clinicians must also rely on clinical judgment or decision rules for obtaining CT scan, regardless of the presence or absence of a skull fracture in US.

In a multicenter, prospective, and observational study, Parri et al. determined the accuracy of POCUS in identifying skull fractures in children younger than 2 years of age with local signs of head trauma and they showed The sensitivity of POCUS for skull fractures was 80 of 88 (90.9%; 95% CI 82.9–96.0) and the specificity for skull fractures was 23 of 27 (85.2%; 95% CI 66.3–95.8). None of the 8 patients with false-negative POCUS results had clinically important TBIs. [6].

In a systematic review/meta-analysis conducted by Gordon et al., to determine the operating characteristics of POCUS skull studies in the diagnosis of fractures in pediatric head trauma patients, six studies comprising 393 patients with a low risk of bias were selected. The pooled sensitivity was 91%, and specificity was 96%, resulting in a pooled positive likelihood ratio of 14.4 and negative likelihood ratio of 0.14. Using the weighted prevalence of skull fractures across the studies as a pretest probability (31%), a positive skull ultrasound would increase the probability to 87%, whereas a negative test would decrease the probability of a skull fracture to 6%. To achieve a post-test probability of a skull fracture of ~ 2%, a negative skull ultrasound in a patient with a pretest probability of ~ 15% would be required [7].

Our case report underscores the successful diagnosis of a skull fracture and brain injury in an infant using US. Although the existing body of literature is scarce, US is emerging as a valuable imaging modality for diagnosing skull fractures in infants, providing a radiation-free alternative to CT scans. Further research is warranted to explore the full potential of US in diagnosing various types of head traumas in pediatric patients.

## Supplementary Information

Below is the link to the electronic supplementary material.

Online Resource 1 Point-of-care Ultrasound using a very-high frequency (18-5 MHz) linear array transducer in the acute phase of post-traumatic injury. The misalignment of the cortical bone is coupled with the periosteal bulging and subdural hematoma. Supplementary file1 (AVI 47574 kb)

Online Resource 2 Point-of-care Ultrasound using a very-high frequency (18-5 MHz) linear array transducer in the advanced phase of the bone callus formation. The subdural hematoma is no longer visible. The bone callus appears as a hyperechoic line, similar to the surrounding normal bone cortex, but the underlying reverberation artifact are absent. Of note, the mentioned artifact stops abruptly exactly at the transitional zone from the normal bone cortex to the prominence of the callus. Supplementary file2 (AVI 28463 kb)

## Data Availability

The authors confirm that the data supporting the findings of this study are available within the article and its supplementary materials.

## Abbreviations

CT: Computed Tomography
US: Ultrasonography
ER: Emergency Room
MRI: Magnetic Resonance Imaging
PECARN: Pediatric Head Injury/Trauma Algorithm
POCUS: Point-of-Care Ultrasound
